# Utilisation of rural primary health centers for outpatient services - a study based on Rajasthan, India

**DOI:** 10.1186/s12913-022-08934-y

**Published:** 2023-04-22

**Authors:** Ambey Kumar Srivastava, Rajan Kumar Gupt, Ruchi Bhargava, Rajesh Ranjan Singh, Dinesh Songara

**Affiliations:** 1 Lords Education and Health Society, 159, Santosh Nagar, Jaipur, Rajasthan 302019 India; 2 Lords Education and Health Society, Building No. 24 (3rd Floor), Okhla Phase-III, New Delhi, 110020 India

**Keywords:** Primary healthcare, Primary health centers, Utilisation, Determinants, Outpatient services, Patient preferences

## Abstract

**Introduction:**

Outpatient services are crucial for strengthening primary healthcare and reducing out-of-pocket spending, which has been one of the major causes of impoverishment. So it is also critical to comprehend the people’s preferences in accessing primary healthcare facilities, as government primary healthcare facilities in India are underutilized. The current paper explores the factors that construct the individual’s decision to seek outpatient care in primary healthcare facilities in India’s largest state Rajasthan.

**Methods:**

It was a cross-sectional survey conducted in 72 primary sample units of 24 primary health centers in 11 districts of Rajasthan, India, from November 2019 to January 2020. The study selected 368 households through purposive sampling. Out of 368 households, 460 people reported any illness and 326 reported outpatient visit to any health facility in the last 30 days from the date of the survey.

**Analysis:**

The focus was on analyzing the data in the context of public and private health facilities to understand the factors influencing people’s choice to access outpatient services. The principal component analysis is used to understand the relationship between facility preparedness and OPD uptake. Also, multivariate logistic regression is applied to assess the significant predictors in using primary health facility services.

**Result:**

Except for the 29% of patients who received no care, the proportion of patients attended public health facilities was 35%, and the rest were utilizing private health facilities. Those who sought care at PHCs were mostly over 45 years age, non-literate, and from the lowest wealth quintile. Logistic regression suggests that people belong to upper wealth quintile (OR = 0.298; 95% 0.118–0.753) are less likely to visit PHCs for treatment. Also, increase in distance of PHC (OR = 0.203; 95% CI 0.076–0.539) reduces the likelihood of their visit outpatient care. People are 9.7 times (OR = 9.740; 95% CI 2.856–33.217) more likely to visit a PHCs that are better equipped in terms of human resources, equipment, and medicine.

**Conclusion:**

The uptake of PHCs depends on several factors, which should be considered to ensure that all segments of society have equitable access to them. Through improved accessibility and quality of service, PHCs can be made more appealing to the larger population.

## Introduction

The decision to seek health treatment and where to seek [1, 2] are the two primary questions that every patient would always prefer to have answered before going to the OPD (Out Patient Department) care, which is considered to be the patient’s first point of contact with the hospital [3]. Patient satisfaction with outpatient department services has been studied at length in both qualitative and quantitative ways [4, 5]. The primary healthcare services, provided through primary health centers (PHCs) can play a critical role in bringing down the OPD care cost as it can address 80% of people’s health needs [6] and the literature review suggests that the economically vulnerable spend more on OPD, which contributes to their impoverishment [7].

India has an elaborate network of nearly 200,000 Government Primary Health Facilities (GPHCFs), which have enormous scope to increase the utilization for outpatient services [8] because they are grappling with various challenges. Several primary health facilities are poorly equipped and have an inadequate infrastructure. 60% of PHCs in India have only one doctor while about 5% have none [9]. According to the Rural Health Statistics report 2020–21, 21.1% of the sanctioned posts of female health workers at sub centers (SCs) and PHCs, 41.9% posts of male Health workers, 64.2% posts of health assistants, and 21.8% of the sanctioned posts of doctors are vacant in PHCs []. PHCs, which cater to a limited percentage of people’s needs, are bypassed, and fail to form the heart of people-centered integrated care [11]. Several reasons have been identified in previous Indian studies for inadequate access and bypassing the public primary healthcare facilities which include, the health needs of patients, high absenteeism of healthcare staff, low service quality, extensive travel distances, prior experiences, intensity, and duration of ailment [12–16]. As per the NSSO, roughly 20% of urban and 28% of rural households identified financial restrictions as a limiting factor in not seeking medical care for an ailment [17]. Primary healthcare accounts for 52.1% of India’s current public expenditure on health [18] and there is a need to boost public health spending because an increase in public spending to 2.5–3% can substantially reduce OOPE from the current level of 65 to 30% [19]. Under target 3.8, achieving universal health coverage is one of the sustainable development goals of India required to strengthen primary healthcare [20].

The current paper explores the factors that construct the individual’s choice to seek treatment in the catchment population of selected primary healthcare facilities in the state of Rajasthan, which is geographically the largest state of India and has poor socio-economic and health indicators. The infant mortality rate and maternal mortality ratio of the state is 32 [21], and 141 [22], respectively. In the state, the prevalence of hypertension among women and men aged 15 and above is 15.3 and 17.9, respectively, which is lower than the national average of 21.3 in women and 24.0 in men. Women and men aged 15 and above have random blood glucose levels (> 160 mg/dl) of 2.8 and 3.3, respectively, which are again much lower than the national average of 6.3 in women and 7.1 in men [23]. In the report “Healthy States, Progressive India” developed on the basis of indicators related to health outcomes, key inputs, and processes, Rajasthan is placed at the 16th position among the 19 big states of India and rated as the weakest performer [24]. Hence, the role of primary health facilities in the state becomes crucial for strengthening of primary healthcare and improving health outcomes, which calls for an in-depth analysis of the factors that influence the behaviour of individuals seeking healthcare at primary health facilities.

## Methodology

### Research design

The study used data from the ‘Out of Pocket Expenditure’ (OOPE) survey which was done as one of the components of the Rapid Health Survey (RHS) conducted in rural PHCs in Rajasthan, India, from November 2019 to January 2020. It was a cross-sectional study, covering randomly selected 72 primary sample units (PSU) in 24 rural PHCs for collecting data on maternal and child health issues, communicable and non-communicable diseases, and out-of-pocket expenditure. Two villages from each PHC were selected using the Probability Proportion to Size (PPS) method.

### Study geography

The PSUs were chosen from 24 PHCs spread over 11 districts of Rajasthan, India, in three different geographies, namely Churu and Jhunjhunu in north Rajasthan; Udaipur, Rajsamand, Dungarpur, and Pratapgarh in Southern Rajasthan; and Sawai Madhopur, Kota, Bundi, Jhalawar and Baran in Southeast Rajasthan. There were five PHCs in the north, 13 PHCs in the south, and six PHCs in southeast Rajasthan. The average population per PHC was 18,226. These 24 PHCs were being managed by the NGO Lord Education & Health Society under a Memorandum of Understanding signed with the Government of Rajasthan to strengthen their services under a public-private partnership arrangement.

### Sampling of respondents

Looking at the available resources and time, the sample number of households was kept at 5 from each PSU. The selection of households was done through purposive sampling by interacting with people in the village. Thus, a total of 368 households were selected for the OOPE study. Out of 368 households surveyed a total of 460 people reported any illness and 326 people reported visits for OPD care in the last 30 days from the date of the survey. A facility preparedness assessment was also completed for all 24 PHCs and purposefully selected a sub-center within each PHC.

### Survey quality

The quality of the survey was ensured by developing protocols and a guidebook. Quality control measures such as back checks, spot checks, and field check tables were also been introduced. Ethical approval of the study was taken from an independent Institutional Review Board (IRB). To respect and maintain privacy and confidentiality during the interview process, the male and female investigators were recruited into the team to ensure that women respondents would be interviewed by female investigators, whereas male respondents by male investigators. The questionnaires developed in English were also translated into Hindi so that investigators could better communicate with the study’s participants. The study team had around 15 days of classroom training to assure high-quality data. Tools developed on CAPI (Computer-Assisted Personal Interviews) were pretested and the team was imparted training on the software.

### Data analysis

The facility preparedness index was developed using principal component analysis (PCA) to investigate the connection between facility readiness and OPD utilisation. The Facility Readiness Index takes into account several different aspects of a healthcare facility, including its human resources (medical and paramedical staff of PHCs), infrastructure, medicine supply, and laboratory tests. A two-point Likert scale from 0 to 1 is used to categorize the facility preparedness index, where 0 means “no” and 1 means “yes.” The reliability coefficient (Cronbach’s alpha) for the scale was 0.78, indicating that it is reliable. Based on the index score, the quality of the facilities was rated as either low, medium, or high.

Bivariate analysis was done using STATA version-15.1 to understand the distribution of treatment care by background characteristics. The effect of individual, community, and facility-level determinants on PHC accessibility in the 30 days before the survey was assessed using multivariate analytic logistic regression. For logistic regression, outpatient care at PHC was used as the dependent variable to determine the associated factors at the individual, community, and facility levels. Those who were going at PHC were coded as ‘1’ and those who were going at other facilities or had not visited any facility were coded as ‘0’. Age group, sex, married status, education, religion, caste, wealth quintile, sickness, community level-distance from village to PHC, and facility preparation were taken as the independent variables.

If Yi is the dependent variable, Xi is a set of explanatory variables, and βi’s are the coefficient, then the logistic regression equation is

$$logit(P)=\log \left(\frac{p}{1-p}\right)={\upbeta}_0+{\upbeta}_1{\textrm{X}}_1+{\upbeta}_2{\textrm{X}}_2+\dots \upvarepsilon$$

Where p predicts the probability and log odds of p and (1– p) provide the odds ratios on the reference category.

## Results

### Profile of respondents

The social profile of 460 patients revealed that almost half of them were 45 years of age or above with 60% male, married (72%) and the majority (44.4%) being non-literate. 98% of the population is constituted by Hindus, with 42% belonging to the OBC group, followed by 27.6% Scheduled Castes (SCs) and 24.3% Scheduled Tribes (STs). The distribution of wealth quintiles was almost equal in all three quintiles, ranging from 32.3% in the lowest to 33.9% in the highest.

### Utilisation of primary healthcare facilities for OPD services

The results are analyzed to understand how the utilisation of facilities differs with the facilities, disease pattern, and socio-economic profile because all PHCs are located in different geography and have different characteristics. Therefore, through analysis, an attempt has been made to understand a comparative picture.

**Fig. 1:**
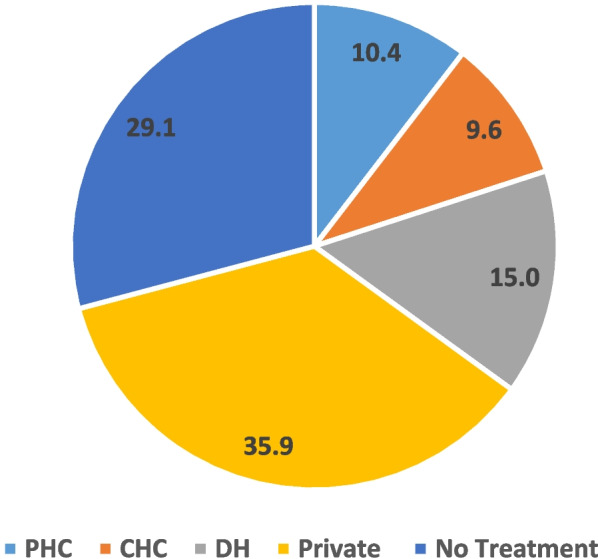
Utilisation by type of health facilities (%), RHS 2019–20

**Fig. 2:**
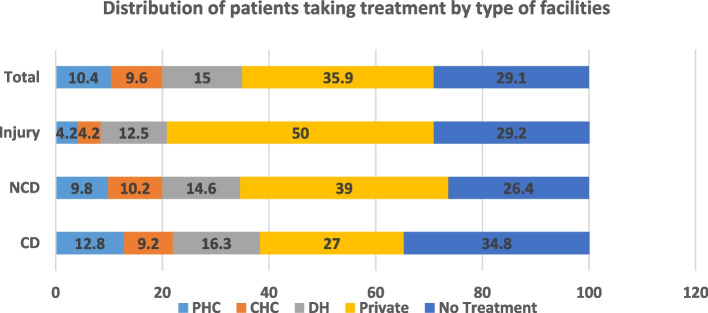
Distribution of patients of CDs, NCDs and injury taking treatment by type of facilities, RHS 2019–20

- **Utilisation by type of facilities**
  The efficient utilisation of primary health facilities is critical to the improvement of service delivery of healthcare system as they will not only alleviate the burden on the secondary and tertiary health care facilities but also reduces beneficiaries’ out-of-pocket expenditure. In rural areas, however limited, but still there are options available to select the health facility for treatment. The analyzed data of respondents reveals that barring 29% of ‘no treatment’ cases, the share of public health facilities, namely PHC, Community Health Center (CHC), and District Hospital (DH) was 35%, and private^1^ 36% (see Fig. 1). In a study conducted on the utilisation of rural PHCs in South India by Sivanandan et al., only 44.5% of individuals visited any health facility of which the proportion of people seeking care at rural PHCs was 70.4% [25]. According to our study, 70.86% of the total 460 patients had received treatment at any health facility, either public or private. Excluding the ‘no treatment’ cases, 14.72% had received treatment at primary health centers. However, within public health facilities this share of PHC was 29.81%, which shows a huge scope to shift the load of higher facilities towards primary health centers in rural Rajasthan.

- **Utilisation of health facilities by type of disease**
  PHCs are the first point of contact to access the primary health services and continuum of care for both communicable and non-communicable diseases. Non-Communicable Diseases (NCDs) show a higher prevalence (64.1%) followed by Communicable Diseases (CDs) at 30.7% and injury at only about 5% in the studied PHCs. In context to facility utilisation, regardless of CD, NCD, or injury cases, a larger proportion of patients were going to private health facilities for treatment than public health facilities (see Fig. 2). In the 30 days before the survey, about 35% of those with CDs, 26.4% of those with NCDs, and 29% of those with injuries did not seek OPD treatment. The disease-wise distribution of public health facilities demonstrates that the share of PHCs is higher in CDs, even though the load is obvious in district hospitals. PHCs outnumber CHCs and DHs by a wide margin, but the data shows that they are currently bearing a disproportionate share of responsibility for treating patients.
- **Utilisation of health facilities by socio-economic profile**
  Primary health facilities are meant for people of all segments of society. However, the variations are found in their utilisation based on different socio-economic characteristics. In the study the analysis in Table 1 reveals that people of age 45 and above were the most likely to seek treatment, while those below 29 were the least likely (56%). Patients aged 45 and older make up nearly half (47.9%) of PHC’s patient population. In contrast, PHC has the highest percentage of people between the ages of 30–45 (29.2%), more than any other facility category. Gender-wise analysis shows that females were less likely to seek treatment in comparison to males. However, among women the PHC uptake is highest among all the facilities. It suggest that women have easier access to PHCs but not to other higher facilities like their male counterparts. Married people reported higher rates of OPD utilisation than those who had never been married or were widowed. There was a higher rate of treatment-seeking behaviour among those educated up to middle and above (72.4%) and it declined as education level declined, with only 59% among non-literate individuals. But those who were going to PHC and to private healthcare facilities for OPD services, the majority were nonliterate. The social composition of respondents shows that the majority of patients taking treatment at district hospitals and private institutions were from the OBC (Other Backward Castes) group, whereas the shares of SC (43.8%) and ST (52.3%) was higher at PHCs and CHCs, respectively.
  The studies shows that poor people are benefited more from primary health care services [26]. Our research also shows that higher proportion of patients (42%) at PHCs belong to lowest wealth quintile, whereas the middle and upper wealth quintiles each account for 29% of patients. Private health facilities have a higher variance in the wealth quintile of patients, ranging from 26.7% in the lowest to 41.8% middle quintile, whereas CHC has a lower variation, ranging from 29.5% (middle) to 36% (highest). At district hospital it ranges from 27% in the richest quintile to 39% in the lowest quintile. The majority of those who did not seek treatment were in the 45+ age group, male (56.7%), and nonliterate but the proportion was almost equal in the lowest and highest quintiles .

**Table 1:** Socio-economic profile of all respondents visiting PHC/CHC/DH and private facilities for OPD care, RHS 2019–20

	PHC (%)	CHC (%)	DH (%)	Private facilities (%)	No treatment (%)	Total (N)
**Age in years (%)**						
Below 29	22.9	20.5	20.3	28.5	32.8	125
30–45	29.2	25.0	20.3	20.0	23.1	103
45+	47.9	54.5	59.4	51.5	44.0	232
**Sex (%)**						
Male	52.1	70.5	71.0	58.2	56.7	277
Female	47.9	29.5	29.0	41.8	43.3	183
**Marital Status (%)**						
Never Married	18.8	15.9	11.6	18.8	27.6	92
Married	66.7	77.3	84.1	72.1	67.2	333
Widow/Divorce	14.6	6.8	4.3	9.1	5.2	35
**Education (%)**						
Non-literate	60.4	56.8	23.2	47.9	41.0	204
Primary	18.8	22.7	43.5	25.5	31.3	133
Middle/Above	20.8	20.5	33.3	26.7	27.6	123
**Religion (%)**						
Hindu	93.8	97.7	97.1	97.6	97.8	447
Muslim/Sikh/Bodh	6.3	2.3	2.9	2.4	2.2	13
**Caste (%)**						
SC	43.8	25.0	13.0	20.6	27.6	112
ST	31.3	52.3	13.0	28.5	24.6	127
OBC	20.8	20.5	69.6	42.4	41.8	193
Other	4.2	2.3	4.3	8.5	6.0	28
**Wealth (%)**						
Lowest	41.7	36.4	39.1	26.7	36.6	156
Middle	29.2	29.5	33.3	41.8	27.6	156
Highest	29.2	34.1	27.5	31.5	35.8	148
**Total (N)**	**48**	**44**	**69**	**165**	**134**	**460**

### Reasons for taking treatment at different health facilities

Knowing about the reasons of people to go at different health facilities not just help to know their perception but also a way to understand their preferences which can be addressed through planning primary healthcare facilities and services. People’s preferences, vary for various reasons, which can also widen the inequalities [27]. Therefore, the respondents were probed on eight different reasons related to personal convenience in seeking treatment, the availability of healthcare professionals, their outlook on the quality of care, and the overall standard of care (Table 2). Analysis resulted into the findings that the quality was the main reason for going to private facilities (59.4%) and availability of essential services (41.8%), while distance has no role to play. Contrary to that, distance (66.7%), free medicine (41.7%), and the availability of essential services (33.3%) were the top cited reasons for preferring primary health facilities. In the case of CHCs too, free medicine (54.6%), availability of required services (38.6%), and quality of services (36.4%) emerged as the key determinants. Regarding district hospitals, the respondents mentioned quality of care (71%), the availability of required services (66.7%), free medicine (42%), and the length of waiting times (42%) as the prominent reasons to visit for outpatient services. The proximity (66.7%) seems to be the only emerging reason in favour of PHCs while among other responses either CHC or DH scores better than PHC. The uptake of CHC and DH was found higher as they are better equipped with human resource, equipment and specialist healthcare services.

**Table 2:** Reasons to visit health facilities for OPD in last 30 days prior to survey, RHS 2019–20

Reasons	PHC (%)	CHC (%)	DH (%)	Private facilities (%)	Total
It is near to our homes	66.7	20.5	5.8	5.5	16.6
Facility timings are convenient to visit	25.0	27.3	31.9	19.4	23.9
Health personnel are often present	18.8	29.6	37.7	22.4	26.1
Waiting time is within 15 minutes	10.4	2.3	42.0	13.3	17.5
Health personnel are attentive and polite	22.9	18.2	34.8	21.8	24.2
Quality of care is good	20.8	36.4	71.0	59.4	53.1
Required services were available	33.3	38.6	66.7	41.8	45.4
Availability of free medicine	41.7	54.6	42.0	10.9	27.9
**Total (N)**	**48**	**44**	**69**	**165**	**326**

### Logistic regression

In the logistic regression of the factors linked to receiving OPD treatment at a PHC in the last 30 days before the survey in Table 3, socio-economic, demographic, and facility-level factors have been proven to influence the odds of visiting a PHC for OPD. It is found that the primary level of education is associated with lower odds of visiting PHC (OR = 0.418; 95% CI 0.163–1.072) when compared to the nonliterate. In case of caste categories, the people from OBC category are much less likely to use PHCs (OR = 0.363; 95% CI 0.150–0.880). Higher (OR = 0.298; 95% CI 0.118–0.753) and middle (OR = 0.364; 95% CI 0.145–0.915) wealth quintile families too, are less likely to seek medical care from a PHC compared to lower wealth quintile households. PHC usage is also be predicted by the distance patients have to travel to get there. The likelihood of visiting a PHC for treatment decreases as the distance between the village and the PHC increases (OR = 0.203; 95% CI 0.076–0.539). Facilities in the higher group, where preparedness in terms of human resources, equipment, and medicine is good, have considerably higher odds of visiting for treatment (OR = 9.740; 95% CI 2.856–33.217) than those in the lower group.

**Table 3:** Binary logistic regression showing factors for taking OPD care at PHCs, RHS 2019–20

Background characteristics	Odds Ratio	[95% Conf. Interval]	
**Age in years**			
Below 29®			
30–45	1.770	0.495	6.326
45+	1.181	0.311	4.492
**Sex**			
Male®			
Female	1.202	0.572	2.525
**Marital Status**			
Never Married®			
Married/other	0.898	0.260	3.098
Widow/Divorce	1.025	0.202	5.197
**Education**			
Non-literate®			
Primary	0.418*	0.163	1.072
Middle and above	0.509	0.178	1.460
**Caste**			
SC®			
ST	1.340	0.515	3.486
OBC	0.363**	0.150	0.880
Other	0.478	0.081	2.807
**Wealth**			
Lowest®			
Middle	0.364**	0.145	0.915
Highest	0.298**	0.118	0.753
**Disease**			
Communicable®			
Non-communicable	0.813	0.378	1.753
Injury	0.261	0.028	2.407
**Distance**			
< 5 km®			
5–9 km.	0.272**	0.122	0.610
10+ km.	0.203***	0.076	0.539
**Facility Preparedness**			
Low®			
Medium	3.584**	1.262	10.177
High	9.740***	2.856	33.217

Reference @, *P* value *** < .001., ** 001 to.050, * .050 to*.10

### Ranking the reasons to visit PHCs

**Fig. 3:**
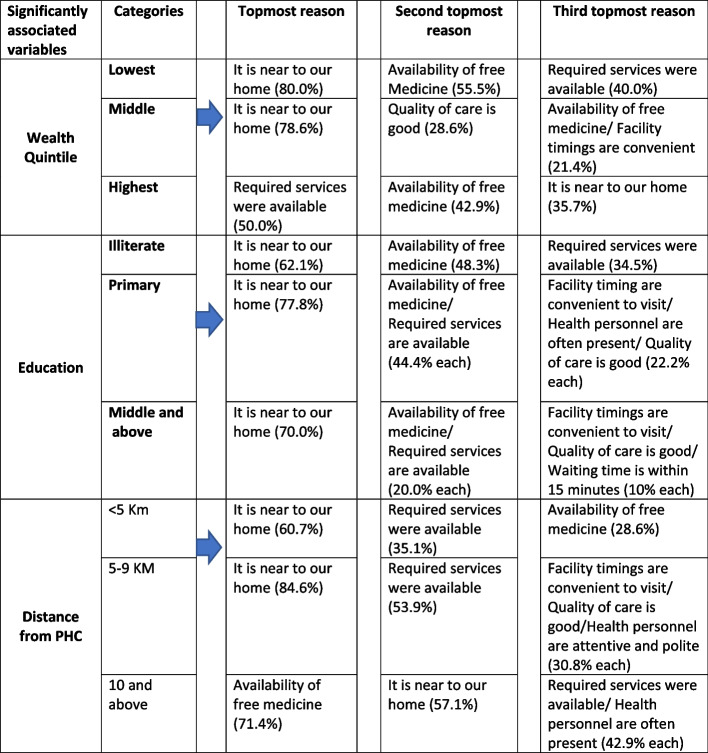
Mapping of top three reasons of the variables which are significantly associated with the OPD visit to rural primary health centers, as per RHS 2018–19

Figure 3 illustrates the mapping of the topmost reasons cited by the respondents based on significantly associated predictors of PHC utilisation, including education, wealth quintile, and distance from PHCs. Here the reasons for visiting PHCs are cross-tabulated with different categories of significantly associated predictors and ranked according to the response percentage. The analysis shows that nearness to a PHC facility was most prominent among all listed factors. The second most commonly reported reasons were ‘free medicine’ and ‘availability of required services’, followed by a ‘convenient time to visit the facility’ and ‘quality of care is good’. Although state has free medicine scheme in all public health facilities but the reasons like ‘availability of required services’ and ‘quality of care is good’ indicates that people are now demanding high-quality primary healthcare. Such preferences require policy level decisions and improved planning of primary healthcare services to enhance the coverage of PHCs.

### Suggestions of the respondents

Suggestions from the patients can be of great help to make the primary health facilities more patient-centered [28] and to improve satisfaction with their services which in turn can also improve the ambulatory services [29]. In our study respondents, who had availed services from primary healthcare facilities in the last 6 months from the date of the survey were asked to suggest ways to improve the PHC services (see Table 4). In the analysis, options with similar interpretations are grouped. A major recommendation was that the staff should increase its reach, which is possible only by having more outreach services. 38.2% respondents suggested reduction in waiting time, followed by improvement in quality care by 36.4%, besides free drug and medicine by 33.6% respondents. 45.6% of patients demand to bring PHCs closer to doorsteps. It shows the need to open more PHCs in areas where people have to travel long distances. The availability of ambulance at facility was suggested by 28.1% of patients. About 41.5% of respondents suggested adding innovative diagnostic equipment, followed by providing more equipment and medicines (33.6%) and a hygienic environment at PHCs (27.2%). Such suggestions reiterate the demand for quality care by patients. This suggestion is further strengthened by 36.4% who have suggested improving quality care at PHCs. 38.2% of respondents demand a reduction in waiting times, indicating they must wait in long queues for their turn. Currently, at PHCs, only 15 tests are eligible per guidelines, and anything beyond that requires a visit to a private or higher public health facility. Furthermore, 32.7% of respondents suggested increasing qualified health personnel availability for improving service quality. Providing staff with soft skills training is recommended to improve their behavior with patients, while the need for female staff stems from the patients’ privacy concerns.

**Table 4:** Suggestions for improving the services at PHC, RHS 2019–20

Suggested Factors		(%) ***N*** = 217
Accessibility	The facility staff should increase its reach	54.4
	Facility should be near	45.6
	The facility should have Ambulance services	28.1
Infrastructure	The facility should have more equipment / Medicine in the facility	33.6
	Hygienic environment in facility should be provided	27.2
	Diagnosis through innovative and advance equipment	41.5
Service Delivery	The health workers should have more home visit	31.8
	Waiting time should be reduce	38.2
	Quality of care should be improved	36.4
	Free treatment and Medicine	33.6
Human Resource and capacity building	Availability of trained staff	32.7
	Facility should have more female staff/doctors	27.6
	The staff needs to be more trained in soft skills	27.2

## Conclusion

This paper attempts to uncover the factors that influence the choices of people when it comes to healthcare facilities. The results conclude that people living near primary healthcare facilities, who are poor, non-literate, have lower education levels, and are in the lower wealth quintile prefer primary healthcare facilities to access healthcare. Well-equipped primary healthcare facilities [30] too, influence people’s perception and their health seeking behaviour. Aside from distance from the facility and facility-level preparation, the other two major predictors support the argument that society’s most vulnerable populations are more likely to use primary healthcare facilities. Therefore, it is very important to make healthcare facilities accessible and acceptable to people from all walks of life. Mapping of the reasons against significantly associated factors, wealth quintile, education, and distance shows that proximity, quality of services, availability of required services, and free medicine are some of the major assessment criteria, which majorly play role in shaping the final decision of people while choosing the healthcare facilities. It is important to note that although this study was conducted with a small sample size, its recommendations call for an increase in the range of services provided by PHCs, a reduction in waiting time, availability of transport facilities, availability of trained staff and sanitation in primary healthcare facilities. These suggestions show that people demand quality healthcare and the necessity of improving the quality of primary healthcare facilities. Strengthened PHCs will not only expand coverage but also impact upon the OOPE. The proportion of persons who do not opt to go for any treatment is also a matter of concern to further deep-dive because it will allow us to understanding their reasons and perspective to seek primary healthcare. Policy-makers also need evidences on reasons for non-utilisation of primary healthcare facilities [31] so that policies and programs can be tailored accordingly. Overall, the effectiveness of primary healthcare facilities is determined by many factors, which are both people and system-driven and a comprehensive approach is needed to improve the utilisation of primary healthcare facilities.

### Limitations of this study

The study is restricted to a smaller geography and its design may not be generalized to the entire state of Rajasthan. There is a need for a larger study with representative sample to verify these findings.

## Data Availability

The data sets generated and/or analyzed during the current study are not publicly available due to the reason that the study was conducted by the LEHS through its own and USAID funds, to understand the performance of primary health centers where it was implementing the program. Also, as an author we would like to know who is accessing the data and for what purpose, since our organization too have data confidentiality and privacy policy. But the data is available from the corresponding author on reasonable request.

## Abbreviations

SC: Sub centre
PHC: Primary Health Centers
GPHCFs: Government Primary Health Facilities
CHC: Community Health Centre
DH: District Hospital
OPD: Out Patient Department
OR: Odds Ratio
LHV: Lady Health Visitors
RHS: Rapid Health Survey
PSU: Primary sample units
IRB: Institutional Review Board
CAPI: Computer-Assisted Personal Interviews
PCA: Principal Component Aanalysis
OBCs: Other Backword Class
NCD: Non-Communicable Disease
CD: Communicable Disease
SC: Schedule caste
ST: Scheduled Tribe
HR: Human Resource
DM: Diabetes Mellitus
TB: Tuberculosis

## Footnotes

1 Private here include traditional and non-traditional healthcare providers, clinics and private hospitals
